# Enhanced performance of lipase via microcapsulation and its application in biodiesel preparation

**DOI:** 10.1038/srep29670

**Published:** 2016-07-18

**Authors:** Feng Su, Guanlin Li, Yanli Fan, Yunjun Yan

**Affiliations:** 1 Key Laboratory of Molecular Biophysics, the Ministry of Education, College of Life Science and Technology, Huazhong University of Science and Technology, Wuhan 430074, China

## Abstract

In the present study, a surface-active enzyme, lipase was immobilized in polyethyleneimine (PEI) microcapsules and then modified with oxidized multiwall carbon nanotubes (MWCNTs). The resulting lipase microcapsules exhibited higher activity and stability, since the activity of microcapsules was 21.9 folds than that of the free counterpart. Numerous interfaces which were created in polycondensation enhanced the performance of lipases. Illustrated by confocal laser scanning microscope (CLSM), it was found that microcapsules, whose barrier properties against molecules with diameter >4.6 nm, were with a semipermeable and porous membrane structure. The lipases preferred to locate in the wall of the microcapsules. The oxidized multiwall carbon nanotubes (MWCNTs) were further added to modify microcapsules, resulting in even higher activity. The nanocomposites were examined by scanning electron microscope (SEM) and zeta-potential analyzer. The results indicated the superior catalytic performances were attributed to the augmented interface and decreased positive charge. Finally, the MWCNTs modified microcapsules were utilized in producing biodiesel with a 97.15% yield and retained nearly 90% yield after running 10 cycles. This approach of microcapsulation will be highly beneficial for preparing various bio-active microcapsules with excellent catalytic performance.

Methyl/ethyl esters of fatty acids, known as biodiesel, are nontoxic, biodegradable, and an excellent alternative for fossil diesel. Biodiesel’s cetane number, viscosity, energy content and phase changes are similar to those of petroleum-based diesel^1^. Transesterification of oils and animal fats for biodiesel by lipases has unparalleled advantages, such as mild reaction conditions, extensive adaptability to crude materials, environmental friendliness and easy downstream-process. Although lipases exhibit many advantages, the high price of enzyme catalysts is the main bottleneck for biodiesel industry. Immobilization is a good solution to this problem because immobilized lipases can be repeatable and more stable than the free one.[Fig f1]

Indeed, enzyme immobilization has been studied for many years in different fields. In literature, the conventional immobilization methods are adsorption, cross-linking, covalent bond and entrapping^2^. Especially, entrapment has drawn increasing attention because the benefits of fastness, cheapness, easy handling and usually involving moderate conditions. Reetz *et al*.^3^ investigated the sol-gel encapsulated lipases, retaining 70% original activity after 20 reaction cycles. The *Pseudomonas cepacia* lipase was entrapped in the hydrophobic sol–gel and the final yield for biodiesel production was about 67%^4^. Yadav *et al*. used calcium alginates as substrates to prepare beads, the obtained biocatalysts were highly reusable with little leaching even after four batches of reuse^5^. However, as known, this bead entrapment of biocatalysts with gel structures, such as sol-gel or calcium alginate, always has the mass transfer restriction, so the performance of the obtained biocatalysts is somewhat limited. Besides, the leakage of enzyme may also lower the performance of enzyme. In addition, extrusion methods may lead to large size particles which increase the mass transfer limitation; though this technique was simple and convenient for a small scale preparation, is not suitable for a large scale application.

As an alternative to the bead entrapment, the microcapsulation is a unique immobilization method that biocatalysts are encapsulated in a thin, semipermeable membrane, which not only permits some specific chemical species to exchange with the medium, but also prevents enzymes from leakage and being contaminated. Actually, microcapsulation is a promising technique being widely exploited for various functions, such as, immobilization biocatalysts^6^, drug carriers^7^, controlled release^8^, and cosmetics^9^. In every case, monomers or prepolymers existing in two immiscible phases are cross-linked to create microcapsules. The encapsulation involves two steps: the formation of stable emulsion and polycondensation occurring at the interface of a microdroplet. Then, the active ingredients are caged in the microcapsules. The micro-level and thin membrane facilitate the decrease of the mass transfer limitation. Moreover, the emulsification step before condensation is well fit for an industrial scale.

The most worth mentioning is that lipases are sensitive to the interface. With the existence of an oil-water interface, the lipase will convert into a lid-open conformation to allow substrates to access to its active site(s) (the open/close conformation is controlled by a lid formed by a surface loop in most lipases). The emulsification step provides numerous microdroplets which contain huge space at the interface. Lipases are activated by the oil-water interface, and therefore, higher activities are obtained. Liu *et al*.^10^ reported that lipases immobilized on magnetic nanoparticles in reverse micelles system would increase the activity-recovery to 382% compared with 29% in aqueous phase. It was because reverse micelles system provided more interface than water phase. The reverse micelles system and the emulsions system here both belong to W/O emulsion. In light of this, we assume that the emulsions step before polycondensation will help activate lipases. To this W/O emulsion, a unique self-assembly of surfactant molecules enclosing a water pool is a host for enzymes. Some researchers add carbon nanotubes (CNTs) into the emulsion system to develop stable self-assembled nanohybrids, resulting in the augmentation of space at the oil-water interface. A surface-activated enzyme, *Chromobacterium viscosum* lipase, localized at the enhanced interface, showed extremely hyper-activation (approximately 2.5 fold) than that in the absence of CNTs^11^. In other previously work, for example, doping of gold nanoparticles within emulsion, was proved to be an elegant strategy to modulate the enzyme activity^12^. Additionally, polyethyleneimine (PEI) polymers are usually used as matrix for lipase immobilization by interfacial polycondensation with sebacoyl chloride. The process is shown in Supplementary Fig. 1. PEI molecules are terminated with amines which are easy to react with the cross-linking agent and for further modification. However, the strong positive charges of amines will cause harm to lipase proteins. Toxicity of amine terminated PEI or dendrimers is caused by the strong electrostatic-interaction between the negatively charged protein and positively charged polymers. It would be intriguing to see if carbon nanotubes were added to decrease the toxicity of PEI, and how the activity of microcapsules would change.

Immobilized lipases have been applied in many fields, such as food industry and biopharmacy, however, one of the most of application is biodiesel preparation. Transesterification of oils and animal fats into biodiesel by lipases has unparalleled advantages, such as mild reaction conditions, extensive adaptability to crude materials, environmental friendliness and easy downstream-process. The most important merit is that microcapsules can be repeatable and more stable than the free one for industrial production. In light of this, the prepared microcapsules lipases are used to catalyze transesterification for biodiesel preparation with soybean oil.

In short, this study investigated the immobilization of lipases by microcapsules. Some specific characteristics related to microcapsule lipases were further studied, including the activity of the immobilized lipases, the morphological characters both in bright and fluorescent fields, size distribution, permeation of microcapsule membranes, and catalysts distribution within microcapsules. Moreover, in order to further improve the performance of microcapsule lipases, multi-walled carbon nanotubes were added to modify them. Finally, the prepared microcapsule lipases were tested by catalyzing transesterification for biodiesel production from soybean oil.

## Results

### Microcapsules for lipase immobilization

**Figure 2:**
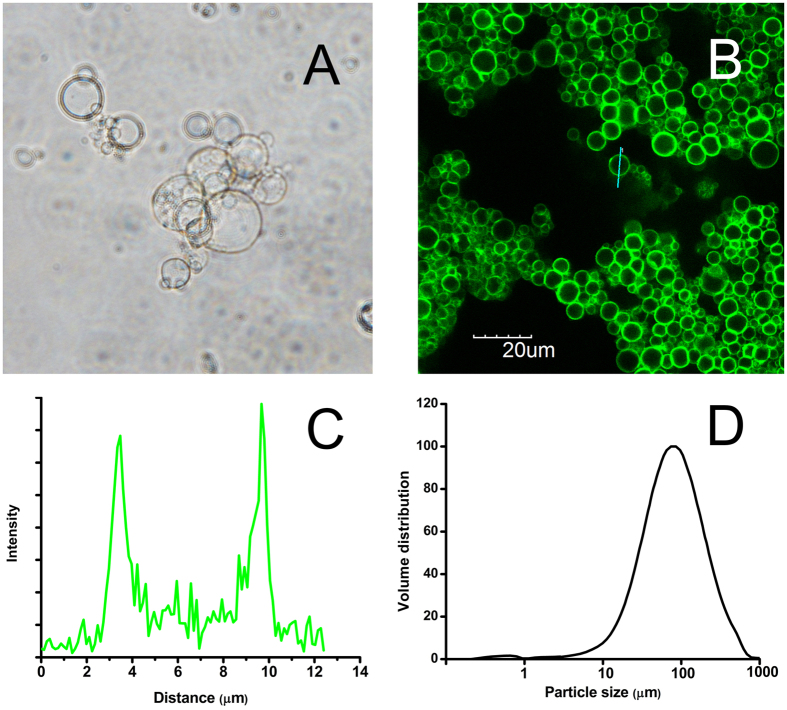
Lipase microcapsules formed by interface polymerization. (**A**) Bright field optical microscopic magic of the emulsion prepared from the mixture 5% PEI and aqueous BCL (100 mg/ml, 200 μL). (**B**) Confocal florescence image of the lipase microcapsule. Aqueous solution of FITC labeled PEI was emulsified for preparing microcapsules with agitation speed of 12000 rpm for 2 min. (**C**) Fluorescence intensity profile along with the line in CLSM image. (**D**) Size distribution of microcapsules.

In this experiment, microcapsules were prepared by the interfacial polymerization in an emulsion consisting of cyclohexane and aqueous lipase (100 mg/mL). According to Fig. 2A, the bright image was obtained by the inverted phase contrast microscope. Many transparent and smooth bubbles aggregated together. Figure 2B showed a more precise observation from Confocal laser scan microscope (CLSM), the green signal on the image came from the PEI tagged with fluorescein isothiocyanate (FITC). The size of an individual spherical particle was determined to about 10 μm in diameters. Nevertheless, from the light scattering evaluation it revealed that the individual particle with a size distribution between 10–100 μm because of aggregation (Fig. 2D).

Lipase from *Burkholderia cepacia* (BCL) was immobilized by microcapsulation. The results showed that while the specific activity of free lipases was 3,431 U/g-protein, and the BCL microcapsules reached 75,104 U/g-protein. The microcapsulation procedure had apparent effects on improving the activity of lipases because the activity recovery was 2,188%.

**Figure 3:**
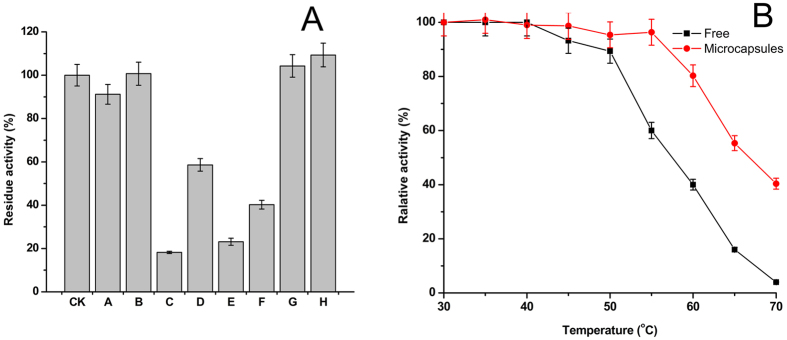
The stability of microcapsules. (**A**) Tolerance to organic solvents where A, B, C, D, E, F, G and H meant methanol, ethanol, propyl alcohol, sopropanol, butanol, isobutanol, acetone and buffer. (**B**) Thermal stability of microcapsules in various temperatures.

As shown in Fig. 3A, the properties of endurance against polar organic solvents were examined. Lipase microcapsules were mixed with polar organic solvents for 4 h and subsequently assaying activities. The residual activities were over 90% for methanol, ethanol and acetone. Nevertheless, the propyl alcohol, butanol and isobutanol caused enormous harm for the immobilized lipases, especially propyl alcohol, remaining less than 20% original activity. Meanwhile, the thermostability of lipases was also examined (Fig. 3B). When lipases were pretreated with various temperatures for 4 h, the residual activities of the microcapsules exhibited no obvious loss when the pretreating temperature was below 55 °C. On the contrary, activities of free lipases sharply decreased when temperature was over 50 °C.

### Permeation of lipase microcapsules

**Figure 4:**
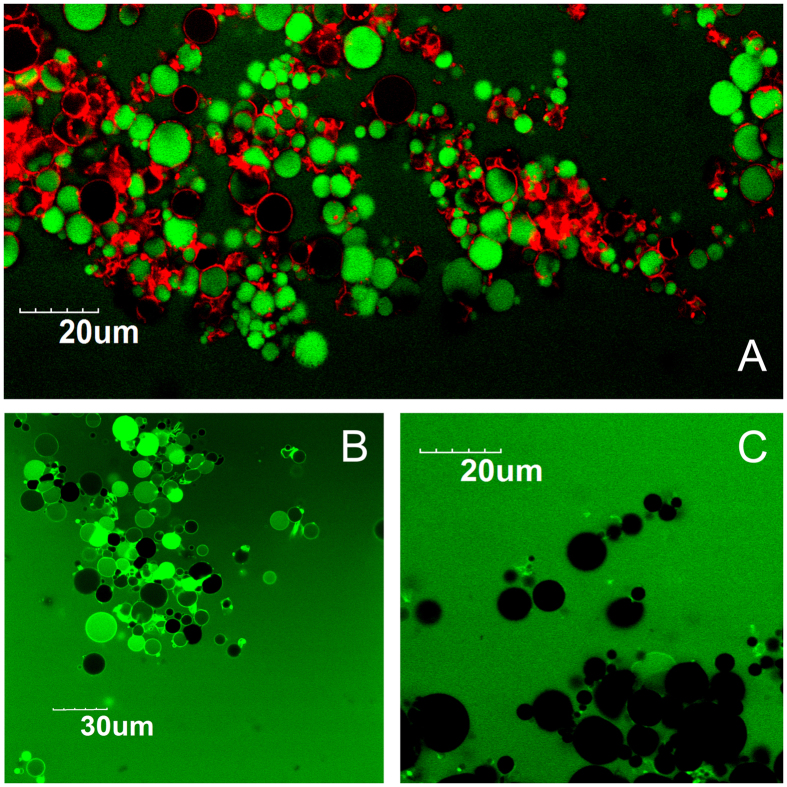
Permeability behavior of lipase microcapsules. (**A**) Confocal fluorescence image of microcapsules in water containing Flu (33 μM) and RhB (33 μM). The fluorescence image was obtained after 30 min by double excitation of 488 nm with bandpass filter (505–530 nm, green) and 543 nm with long-pass filter (560 nm, red). (**B**) Fluorescence CLSM images of microcapsules obtained after 30 min in water containing FITC-labeled dextran (4 kDa). (**C**) Fluorescence CLSM images of microcapsules obtained after 30 min in water containing FITC-labeled dextran (10 kDa).

After being immobilized, lipase molecules were caged in the microcapsules. Permeation property of the lipase-caged microcapsules was further examined. Herein, Fluorescent dye molecules, sodium fluorescein, (Flu) and rhodamine B (RhB) were used as permeants. Prior to adding dye molecules, microcapsules consisting of lipase were prepared, and their ζ potential was also determined. The result showed that surface charge of microcapsules was +40 mV. Equimolar mixture of Flu and RhB in buffer was added. After 30 min, the CLSM images of microcapsules in aqueous dye solution were observed (Fig. 4A). The Flu molecules emitted green luminescence in the 505–530 nm region (excitation wavelength, λex = 488 nm), whereas the RhB emitted red luminescence at a wavelength beyond 560 nm (λex = 543 nm). According to Fig. 4A, the green luminescence of Flu was observed both outside and inside of the microcapsules. In contrast, red luminescence was preferentially observed on the microcapsules wall.

To investigate the permeant diffusivity of microcapsules, FITC-labeled dextran (FITC-dextran) with 4 k and 10 k average molecular weights were prepared as probes. CLSM images of microcapsules soaked in buffer for 30 min after adding FTIC-dextran solution (λex = 488 nm) were obtained. When the FITC-dextran (4 k Da) was added, the fluorescence signal was observed from the inner space of microcapsules (Fig. 4B). Unlikely, In Fig. 4D, the bright field image showed that some microcapsules aggregated. When the FITC-dextran with 10 k was added, none green signal was observed in the capsule aggregate and there was no single microcapsule filled with green signal. The FITC-dextran with 10 k molecular weight cannot penetrate into the inner volume (Fig. 4C).

### Catalyst positioning

**Figure 5:**
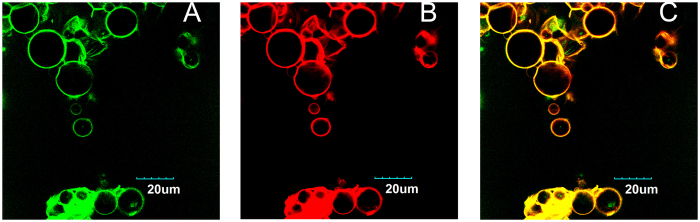
Distribution of protein in the microcapsules. Fluorescence CLSM images of microcapsules which was prepared by aqueous BSA with 2 mg/mL and the agitation speed was 4500 rpm for 2 min. (**A**) Single fluorescence CLSM images of PEI which was tagged with FITC. (**B**) Single fluorescence CLSM images of BSA which was tagged with Texas red. (**C**) The signal of PEI and BSA were overlapped.

To investigate the catalyst location in microcapsules, the bovine serum albumin (BSA) tagged with Texas red was used as the lipase analogue. Tagged BSA and PEI (tagged with FITC) were incorporated in the aqueous phase before polycondensation. The preparation of microcapsules was the same as presented above. As shown in Fig. 5, the red fluorescence revealed the presence of the BSA, which only existed in the wall of the capsules with a high density, and it was superimposed with the green fluorescence related to the FITC-tagged PEI.

**Figure 6:**
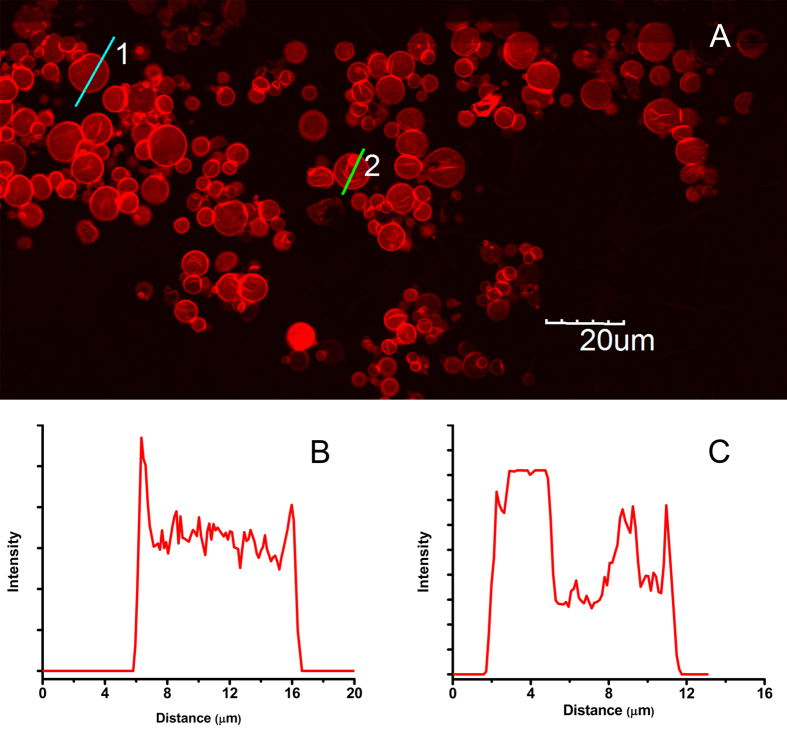
The effect of high BSA concentration on protein distribution in the microcapsules. (**A**) Fluorescence CLSM images of microcapsules which was prepared by aqueous BSA with 20 mg/mL. (**B,C**) Fluorescence intensity profile along with the lines in CLSM image.

The low fluorescence intensity in the core of the microcapsules was caused by the low concentration of BSA (0.2 mg/mL) used in experiments. To evaluate the effect of concentration on proteins distribution, 100 folds concentration (20 mg/mL) BSA was used to prepare microcapsules for the CLSM observation. Figure 6 presented the uniform distribution of proteins inside the microcapsules. Although the red signal was observed both in the wall and in the core of the capsules, the fluorescence intensity was slightly higher in the wall.

### Carbon nanotubes modified microcapsules for lipase immobilization

**Figure 7:**
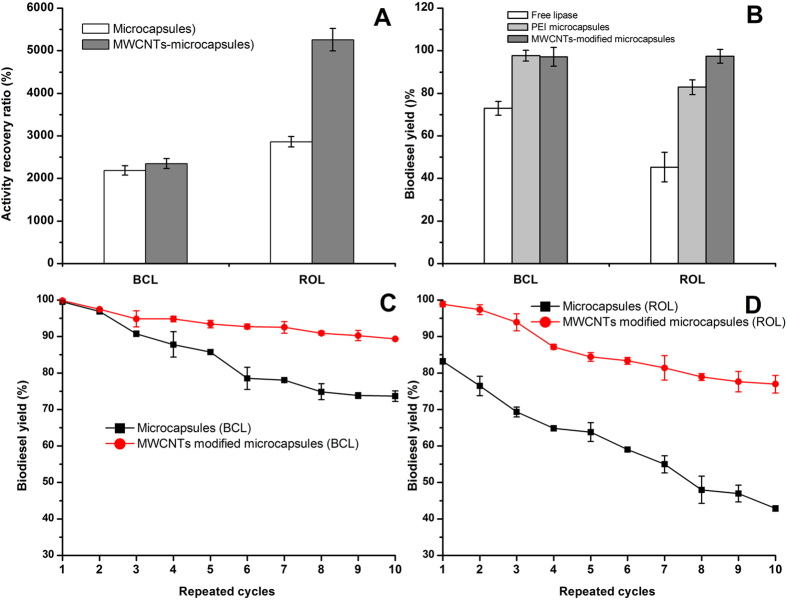
(**A**) The effect of MWCNTs on the activity recovery ratios of BCL and ROL microcapsules. (**B**) Transesterification catalyzed by BCL and ROL. [Conditions for BCL were oil, 2.19 g; enzyme loading, 1 g; molar ratio of ethanol/oil, 4:1; moisture concentration, 5 wt% (based on the oil weight); reaction temperature, 40 °C; stirring rate, 200 rpm; reaction time, 12 h], [Conditions for ROL were oil, 2.19 g; enzyme loading, 1 g; molar ratio of ethanol/oil, 4:1; moisture concentration, 30 wt% (based on the oil weight); reaction temperature, 45 °C; stirring rate, 200 rpm; reaction time, 48 h]. (**C,D**) Reusability of microcapsules and modified microcapsules. The conditions were followed with **B**.

Carbon nanotubes (CNTs) were added to modify lipase microcapsules. The production of multiwall carbon nanotubes (MWCNTs)-modified microcapsules was detailed below. Firstly, pristine CNTs and oxidized CNTs were both examined by the X-ray photoelectron spectroscopy (XPS) with which the extent of oxidation could be evaluated. In Supplementary Fig. S1, a typical oxygen element peak was observed in the oxidized CNTs and the oxygen content of pristine CNTs and the oxidized ones were 1.03% and 18.85%, respectively. Then, 0.05 g CNTs was added to react with PEI. Finally, the original microcapsules and modified ones were compared by assaying their esterification activities. In Fig. 7A, the MWCNTs modified microcapsules showed higher recovery activity for both BCL and lipase from the *Rhizopus oryzae* (ROL). Especially for ROL, the recovery activity reached 5,258%, which was almost two-fold compared to the original microcapsules (2,961%).

### Transesterification for biodiesel preparation

Microcapsules were employed to catalyze biodiesel synthesis with soybean oil to evaluate its practical application. In Fig. 7B, the free lipase, microcapsules and MWCNTs modified microcapsules were compared. The results showed that the biodiesel yield of BCL microcapsules and ROL microcapsules separately were 97.65% in 12 h and 82.86% in 48 h, both of which were much higher than those of their free counterparts (72.98% and 45.32%). Furthermore, the performance of MWCNTs modified microcapsules was even better than that of original microcapsules. For the BCL and ROL, the final biodiesel yields of modified immobilized lipases were respectively 97.15% and 97.38%.

Reusability is the most important issue for the immobilized lipases in industrial application. The reusability of microcapsules was investigated with results presented in Fig. 7C,D. For BCL, while the microcapsules remained 73.66% biodiesel yield, the MWCNTs modified microcapsules retained 89.32% yield after running 10 cycles. For ROL, the microcapsules and MWCNTs modified microcapsules respectively remained 42.98% and 76.94% yield after running 10 cycles.

## Discussion

Microcapsulation is regarded as an excellent way for immobilization because the immobilized matrix is expected to be repeatable, efficient and stable in the harsh environment. Microcapsules were defined as mono or multinuclear materials enclosed by a coat or membrane, in which active ingredients were incorporated into small size multi-particulate units^13^. These initial experiments have shown that lipases can be immobilized as microcapsules.

Actually, the method of microcapsulation has developed for many years. In the year 1931, the micro-encapsulating procedure for pharmaceuticals was published by Bunger Burg De Jong & Kars involved in the preparation of gelatine spheres^14^. Methods of microcapsulation including bulk polymerization, suspension polymerization, emulsion polymerization, interfacial polymerization, and layer by-layer deposition and so on, are chosen by the monomer types and experimental purposes, For example, Zhang *et al*. used layer by layer deposition to prepare microcapsules (catalase from bovine liver)^15^, CaCO_3_ was used as the core material for microcapsules, and then coated the core with polydopamine (PDA) and PEI in this order. Subsequently, titania (Ti) as the mineral layer was deposited on the surface of PDA-PEI-CaCO_3_. By washing away the CaCO_3_, polymer-inorganic microcapsules were obtained, and the hybrid microcapsules caused a varied activity (42–73 U/mg). Nevertheless, this method is not the most suitable one for lipases immobilization, interfacial polymerization, instead of layer by layer deposition, is more suitable for lipase immobilization. The reasons are: Lipases are sensitive to the interface are different from other hydrolytic enzymes. Lipases tend to be adsorbed by the oil/water interface and then be activated by the interface, resulting in higher activities. It was because the lipase would convert into a lid-open conformation to allow substrates to easily access to its active site(s) with the existence of an oil-water interface^11^. In consideration of surface-active, it is necessary to utilize the process of interfacial polycondensation which provided numerous interfaces to activate lipases and spontaneously make lipases into an immobilized form. This interfacial activation in polycondensation was mainly attributed to improvement of BCL whose activity recovery was high as 2,188%. Liu *et al*.^10^ have also prepared hyperactivated lipases in W/O emulsion, with the activity-recovery increasing from 29% in aqueous to 382%. All these results demonstrated that microcapsulation via interfacial polycondensation was an efficient method for lipase immobilization.

Moreover, materials for microcapsule were abundant, such as protein^16^, polymide^17^ and PEI, especially for PEI, it could be directly used as monomer to prepare microcapsules, or as the adhesive molecules to connect with various layers to form multilayer microcapsules^15^, and as glue to fix enzymes on paper substrate to immobilize lipases^18^. PEI was more suitable than polyamides as monomer to prepare microcapsules to immobilized lipases. It was because during the polycondensation of polyamides, a strong diamine base and a dichloride would solubilize in the aqueous solution, leading to a decline of enzyme activity^17^. The PEI had incomparable advantages over the polyamide because it caused less reduction in enzyme activity during interfacial polymerization due to the reactive nature of the membrane formation process.

The microcapsulation permits control of particle size. PEI microcapsules with about 10 μm in diameters were obtained. Usually, the particle size of microcapsules was less than 200 μm. The particle size was dramatically influenced by the emulsification agitation speed, surfactant and emulsion form^13^. The parameter of emulsification agitation speed was the most important one when other parameters were similar. With a higher agitation speed provided, a higher break up force would generate smaller microemulsion. Upon halving the agitation speed, the particle size shifted to about 20 μm.

Lipase microcapsules exhibited strong endurance against polar organic solvents and high temperature. PEI contained a polymerizable group that would yield a cross-linked shell which would protect lipases from the high temperature and organic, while the free lipases were much sensitive to the high temperature solvents. This shell property was beneficial for further application, such as biodiesel production in which methanol/ethanol was as substrates to react with lipases.

The permeation of lipase microcapsules were examined by dyes as probes. Anionic molecules of Flu had permeated into the cationic microcapsules instead of being strongly adsorbed to the PEI layer. In contrast, red luminescence was preferentially observed on the microcapsules wall, which revealed that there was a strong tendency of adsorption of zwitter ionic RhB. To investigate the permeant diffusivity of microcapsules, FITC-labeled dextran (FITC-dextran) with 4 k and 10 k average molecular weights were prepared as probes. It was well known that the stoke radius of dextran molecules with 10 k molecular weight was 2.3 nm^19^. Therefore, if the FITC-labeled dextran cannot permeate into the microcapsules, which suggested the barrier property against molecules was with diameter >4.6 nm. The average diameter of the lipase protein was above 5 nm^20^, much bigger than the hole of microcapsules. That is, the leakage of lipases in microcapsules will be prevented due to the smaller holes of microcapsules. During physical immobilization, catalysts leakage is a common phenomenon which leads to dramatically decline in the performance of the immobilized enzyme. In consideration of an industrial production, this immobilization method should be more efficient to limit losses and make full use of the expensive biocatalysts. Therefore, the microcapsulation caging lipases within the semipermeable and porous membrane structure would bind the catalysts inside but allow substrates to exchange in/out of the microcapsules.

BSA was used as lipase analogue to explore the catalyst distribution in microcapsule. Fluorescence results demonstrated that protein located in the surface and internal part of the membrane. The strong electronic interaction between BSA and PEI can be involved to explain the distribution of proteins; this interaction was well known and widely used for the delivery of long-term peptide and protein drugs^21^. Other researchers have compared the PEI coated and conventional carriers, and found that proteins could be easily absorbed onto the carriers coated with PEI^22^. It was because as that proteins with a negative charge interacted with PEI-coated carriers that had a higher amount of positive charges, leading to the strong absorption. This also supported our results. When the concentration of BSA increased to 20 mg/mL, although proteins were not only mainly localized both in the wall of the capsules, but also inside of the capsules. Significantly, the lipases concentration practically used in experiments was very low, and the lipase proteins would principally locate in the wall of the microcapsules.

Carbon nanotubes were added to modify microcapsules, resulting in boosted activity. As what has described in the introduction part, carbon nanotubes (CNTs) were added to modify microcapsules nanotubes for two reasons: to enhance the interface of W/O emulsion, and to decreasing decrease the positive charge of polyethyleneimines. Activity of lipases microcapsules was dramatically enhanced with CNTs added into which was coincides with previous works where adding signal carbon nanotubes was regarded as an efficient strategy to improve the lipases activity in reverse micelle^11^. This phenomenon was presumably due to augmentation of overall interface in the newly developed self-assembled nanohybrid system. To further explain it, the emulsification step provided many microdroplets which were just like numerous ‘water pool’. The nanotubes themselves acted as supports, and moieties of them tended to remain toward the ‘water pool’. The CNTs with ‘water pool’ have developed a new self-assembled nanohybrid system whose interface would be enhanced. The interface sensitive enzyme, lipase, localizing at the enhanced interface, showed extremely hyper-activation than that in the absence of CNTs^11^. In addition, the strong positive charges of PEI will damage the lipase protein. Thus, the seducement of positive charges had made great contribution to enhancing the activity of immobilized lipases. There were various protocols for linking carbon nanotube with polymers, such as, ester linkage, amide linkage, nucleophilic addition, cycloaddition, atom transfer radical polymerization (ATRP), reversible addition-fragmentation chain transfer (RAFT), ring opening polymerization (ROP) and so on^23^. Here, polymer chains terminated with amino were attached by amidation reaction with the nanotube surface-bound carboxylic acid groups. Due to active groups on sebacoyl dichloride, this cross-linking agent was able to react with polymer and CNTs at the same time, which meant that CNTs were spontaneously added into the microcapsules during interfacial polycondensation. Similarly, a thin film composite (TFC) membrane modified with CNTs was also prepared by interfacial polycondensation. CNTs, m-phenylenediamine (MPD) in water phase and trimesoyl chloride (TMC) in hexane phase, had been utilized to form a thin film composite (TFC) membrane^24^. Interestingly, the surface potential of the PEI microcapsules (+43.6 mV) decreased to +17.5 mV after modification with MWCNTs. It revealed that the adding of CNTs could successfully decrease the positive charges of microcapsules, leading to the improvement of lipase activity. Indeed, through combination of CNTs with PEI to change the surface charges and thus reducing the electrostatic damage on bio-catalysis were feasible in literature, for examples, Shen *et al*. investigated the PEI-mediated functionalization of MWCNTs and their cytotoxicity *in vitro*^25^. PEI was covalently bonded to oxidized CNTs through amide bond formation. The amine grounds of PEI on the surface of CNTs were further modified with acetic anhydride or succinic anhydride to obtain neutral or negative surfaces charges, respectively. The subsequent vitro cytotoxicity assays on both FRO cells (a human thyroid cancer cell line) and KB cells (a human epithelial carcinoma cell line) showed that neutrally and negatively charged polymer-functionalized CNTs were nontoxic to both cell lines at a high concentration, whereas positively charged composites were toxic to FRO cells at a low concentration. Cao *et al*. compared the effect of three different functionalized CNTs in polymerase chain reaction (PCR), namely, the positively charged PEI-CNTs, neutral PEI-CNTs modified with acetic anhydride and negatively charged PEI-CNTs modified with succinic anhydride. The results indicated that the positively and negatively charged PEI-CNTs could significantly enhance the specificity and efficiency of an error-prone two-round PCR, whereas neutral PEI-CNTs had no such effect^26^. Hu *et al*. also reported the PEI-modified single wall carbon nanotubes (SWCNTs) were used as substrate for neuronal growth with reference to its good biocompatibility^27^. Based on these examples, it suggests that changing the surface charge of PEI-CNTs can help improve the biocompatibility of the materials for a variety of biocatalytic applications. Similarly, strong positive charge would cause great harm for the lipase protein, which could be destroyed by amine terminated PEI through the strong electrostatic-interaction between itself and positively charged polymers. Adding of CNTs could successfully decrease the positive charges of microcapsules, and thereby improve the lipase activity. In conclusion, the improvement of lipase microcapsules could be mainly attributed to the reduction the surface charge of PEI-CNTs in this study. Moreover, according to the scanning electron microscope (SEM) images (Supplementary Figs S2, S3), we found that some CNTs were aggregated and embedded into the polymers, instead of outside of the polymers. The augmented interface and change of charges may be main reasons for the hyper-activation of lipase activity and further experiments may be required to find additional reasons for the improvement.

Immobilized lipase was used to catalyze transesterification for biodiesel preparation with a 97.15% yield, and it retained nearly 90% yield after running 10 cycles. One of the most important applications of lipases is to catalyze esterification/transesterification for biodiesel preparation in mild conditions. Biodiesel has drawn increasing attention as a biodegradable and renewable fuel with lower exhaust emissions, such as CO, HC and SOx^28^. It is usually produced by esterification of fatty acids or transesterification of oils/fats with short chain alcohols. Lipases microcapsules and the CNTs-modified microcapsules both obtained higher biodiesel yields than those of free lipases. It was because the activity of lipase had considerately boosted through interfacial activation, and the microcapsules provided the shell to protect lipases protein from polar solvents, such as methanol and ethanol. Furthermore, the performance of MWCNTs modified microcapsules was even better than that of original microcapsules. This tendency was in accordance with the research in esterification activity, where the esterification activity of MWCNTs-modified microcapsules was significantly better than that of the original capsules. The MWCNTs might decrease the PEI’s damage on lipase proteins, resulting in the improvement of biodiesel preparation. Significantly, comparing with the other works about enzymatically catalyzed biodiesel, it found that the PEI microcapsules showed better performance over other immobilized methods, for examples, BCL was immobilized on hydrophobic magnetic particles to prepare biodiesel with 70% yield within 12 h^29^; biodiesel was produced from *jatropha* oil, catalyzed by immobilized BCL on attapulgite, which obtained 94% yield in 24 h^30^. This study preliminarily confirmed that PEI microcapsules were a favorable method which improved the efficiency of lipase and achieved higher yield of biodiesel in shorter conversion time. When it refers to reusability, it is well known that immobilization is able to enhance the lipase stability and allow for its recycle^31^. Either of the microcapsules and the MWCNTs-modified microcapsules exhibited good stability in biodiesel preparation, yet the MWCNTs modified ones were even better. It assumed that MWCNTs might help maintain the structure of microcapsules and therefore minished the leakage of lipases, leading to a higher yield (89.32%) after several cycles. As an illustration to this, the lipase microcapsules would be washed by cyclohexane and water after each preparation batch in this study. If the immobilized matrix was broken, lipases in the microcapsules subsequently would be cleaned away. The loss of lipases had impaired the biodiesel yield of the next batch. However, the WMCNTs might play an important role in maintaining the structures of microcapsules. As reported in literature, CNTs made contributions to increasing the strength of composites. Olek *et al*. studied the effect of Bamboo CNTs on PEI and found the strength of nanocomposites increased 1,500%^32^, meanwhile, Mamedov *et al*. also found when PEI was grafted with the oxidized CNTs, the strength of hybrid boosted 2,700%^33^. Nevertheless, although microcapsules and MWCNTs-modified microcapsules exhibited good stability, all of the immobilized lipases started to decrease to various extents in yield after 10 cycles. In fact, the phenomenon that immobilized lipase drops gradually with the increase of repeated cycles is unavoidable in all literature. There are three main reasons to illustrate this drop. First of all, the conformation of lipase is maintained by some ‘necessary water’ on the surface of protein. ‘Necessary water’ is easily replaced by polar solvents, such as methanol and ethanol. In the process of biodiesel production, ethanol (as substrates) with poor solubility in feedstock oil may gradually inactivate lipases and shorten its operational life^34^. What is worse, the byproduct of glycerol which adsorbs on the surface of the immobilized lipases impedes substrates to contact with lipases. Most importantly, the leakage of lipases greatly decreases the effect of immobilized lipases. The mechanical force broke a portion of immobilization carriers in the process of continuous reaction. Due to the breakage of carriers, some lipases will leakage and be wash away. In conclusion, the transesterification reaction catalyzed by the MWCNTs modified microcapsules was promising not only for the batch reaction but also for the continuous bed reactor.

In summary, polyethyleneimines microcapsules have been successfully used as matrix for lipases immobilization and then modified with oxidized WMCNTs. The results indicated that PEI microcapsules were an effective matrix for lipase immobilization. The distribution of lipase and permeation of the microcapsules further confirmed. The oxidized WMCNTs modified microcapsules exhibited even better. Lipases immobilized in PEI microcapsules and the MWCNTs modified microcapsules were used to catalyze biodiesel preparation, showing high conversion yields and better operational stability. The study demonstrated that microcapsules immobilization was a promising strategy, especially in catalyzing transesterification reaction for biodiesel preparation.

## Methods

### Chemicals

Lipases from the *Rhizopus oryzae* (ROL) and *Burkholderia cepacia* (BCL) were purchased from Sigma-Aldrich. Polyethyleneimines with 1800 molecular weight, sebacoyl chloride (SC), cyclohexane, Span 80 and bovine serum albumin (BSA) were brought from Shenshi Chemical Industry. Rhodamine B (RhB), sodium fluorescein (Flu), FITC (fluorescein isothiocyanate), FITC-dextran (4 K and 10 K), and reference standards of fatty acid methyl esters were commercially obtained from Sigma-Aldrich. Other reagents, such as acetone, lauric acid, laurinol, ethanol, isooctane, *tert*-butanol, hexane, Na_2_HPO_4_, NaH_2_PO_4_, and NaOH were of analytical grade and got from Sinopharm Chemical Reagent Co. Ltd (Shanghai, China). Soybean oil was taken from local market and its physical properties are: saponification value 183.8 mg/g, acid value 0.23 mg/g, average mean molecular weight 917. The fatty acid compositions are listed in Table 1. The water used in all the experiments is purified with a water purification system and with a resistivity higher than 18.2 MΩcm. The carbon nanotubes used (CNTs) in the microcapsules are multiwalled with 20–40 nm for bore diameter, less than 5 μm for length.

### Preparation of PEI microcapsules

An aqueous phase consisting of 5 mL PEI (5% w/w in water) and 200 μL lipase solution (100 mg/ml in 50 mM-pH 8.0 phosphate buffer) were emulsified together by a emulsifying machine with 9000 rpm for 2 min in the organic phase of 25 mL cyclohexane with 0.5 mL Span 80 as the surfactant. Alternatively, microcapsules were prepared with the BSA (0.2 mg/mL) for the confocal laser scanning microscope (CLSM) analysis. Additionally, the CNTs were oxidized by the nitro-sulfuric acid to obtain the carboxyl grafted CNTs. For CNTs modified microcapsules, nanotubes dispersed in water were added into the aqueous phase prior to emulsification. Sebacoyl chloride was dissolved in 10 mL cyclohexane firstly, and then added into the emulsion to react for 3 min. The additional volume of cyclohexane was used to stop the polycondensation reaction. After settling for minutes, the supernatant was discarded. Then, the microcapsules were washed by additional volumes cyclohexane and water to remove un-reacted substrates. Finally, the PEI microcapsules were collected by the suction filtration with a Buchner funnel.

### *ζ*-protentials and size distribution measurements

The microcapsules were dispersed in ultrapure water and treated by ultrasonic for minutes. Then, ζ-protentials of microcapsules suspended in ultrapure water were measured by a Malvern Zetasizer Nano-ZS apparatus. The particle size was tested through light scattering particle size distribution analysis (Horiba LA-950).

### Confocal laser scanning microscope analysis

To obtain the appearance of microcapsules and study the location of protein inside the capsules, a CLSM (Olympus FV1000) was used. PEI and BSA were tagged with the fluorescein isothiocyanate (FITC) and Texas red, respectively. 488 nm and 561 nm are the excitation wavelength of the FITC and Texas red. To obtain the PEI-FITC, FITC was firstly solubilized in dimethylsulfoxide (DMSO), with PEI dissolved in phosphate buffer 50 mM at pH 8.0. The FITC solution (25 mM) was then mixed with the PEI solution to react overnight at the room temperature and protect from light. Unreacted FITC was then removed by dialysis for 24 h. Tagging BSA with the Texas red followed the above procedures. The excitation wavelengths were set 488 nm for the FITC-Dextran compounds and sodium fluorescein, and 543 nm for the RhB.

### Lipase activity assay

To assay the activity of microcapsule lipases, the lauric acid and laurinol were used as substrates to explore the esterification activity with a molar ratio of 1:1^35^. One unit of activity (U) was defined as the weight of lipase that catalyzes the conversion of 1 μmol of lauric acid lauryl ester in 1 min at 37 °C. The definition of immobilization parameters including specific activity (U/g protein) and lipase activity recovery (%) were calculated from the equations (1, 2):


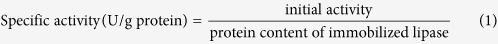



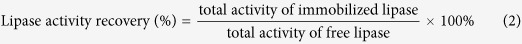


### Transesterification for biodiesel preparation

The microcapsules lipases were employed as catalysts for the practical application, such as biodiesel preparation with soybean oil. The transesterification was conducted in a 50 mL shaking flask under a stirring rate of 200 rpm at 40 °C. The oil was 2.19 g, adding into the reactor firstly, and then a defined amount of ethanol, enzyme and PB buffer were mixed with the oil. After reacting for 12 h, the supernatant was collected by centrifuging in 12,000 rpm. The content of Fatty Acid Ethyl Ester (FAEE) was detected by the gas chromatography with the similar method in literature^35^. The methyl heptadecanoate was used as the internal standard for determining the yield of biodiesel. A GC-9790 gas chromatography is equipped with Agilent INNOWAX capillary column (30 m × 0.25 mm i.d. ×0.25 μm, J&W Scientific, Folsom, CA). The temperature of hydrogen flame ionization detector and injector were both 280 °C. The column was kept at 200 °C for 2 min and increased to 235 °C at a rate of 3 °C/min and then maintained for 1 min in 235 °C. The biodiesel yield (%) was defined as the total FAME content in the conversion oil sample. The biodiesel yield was calculated from equations (3) and (4).


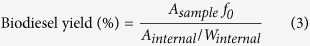



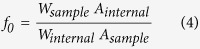


where *A*_*sample*_ is the park area of the free fatty acids in the sample, *f*_*0*_ is the response factor, *A*_*internal*_is the peak area of the internal standard, *W*_*internal*_ is the weight of the internal standard, and *W*_*sample*_ is the weight (g) of the sample.

## Additional Information

**How to cite this article**: Su, F. *et al*. Enhanced performance of lipase via microcapsulation and its application in biodiesel preparation. *Sci. Rep.* **6**, 29670; doi: 10.1038/srep29670 (2016).

## Supplementary Material

Supplementary Information

## Figures and Tables

**Figure 1 f1:**
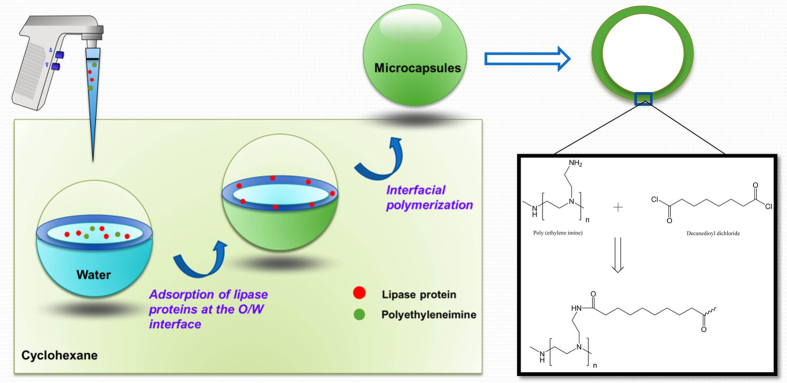
The process of preparing microcapsules.

**Table 1:** The fatty acid constitutions of soybean oil.

Fatty acid	Mass fraction (%)
Palmitic acid (C16:0)	11.0
Stearic acid (C18:0)	23.4
Oleinic acid (C18:1)	53.2
Linoleic acid (C18:2)	7.8
Linolenic acid (C18:3)	4.0
