# Supplementary material for: Enhanced performance of lipase via microcapsulation and its application in biodiesel preparation
Source: Sci Rep. 2016 Jul 18;6:29670. doi: 10.1038/srep29670 (PMC4947930; doi:10.1038/srep29670)
Supplement: Supplementary Information [file srep29670-s1.doc]

**Supplementary information**

**Enhanced performance of lipase via** **microcapsulation and its application in biodiesel preparation**

Feng Su, Guanlin Li, Yanli Fan, Yunjun Yan^*^

Key Laboratory of Molecular Biophysics, the Ministry of Education; College of Life Science and Technology, Huazhong University of Science and Technology, Wuhan 430074, P. R. China

*Corresponding author.

Yun-Jun Yan* (Tel/fax: +86-27-87792213; Email address: yanyunjun@hust.edu.cn)

**Methods**

**S ζ-protentials and size distribution measurements.** The microcapsules were dispersed in ultrapure water and treated by ultrasonic for minutes. Then, ζ-protentials of microcapsules suspended in ultrapure water were measured by a Malvern Zetasizer Nano-ZS apparatus. The particle size was tested through light scattering particle size distribution analysis (Horiba LA-950).

**Supplementary Fig. S1.**

**
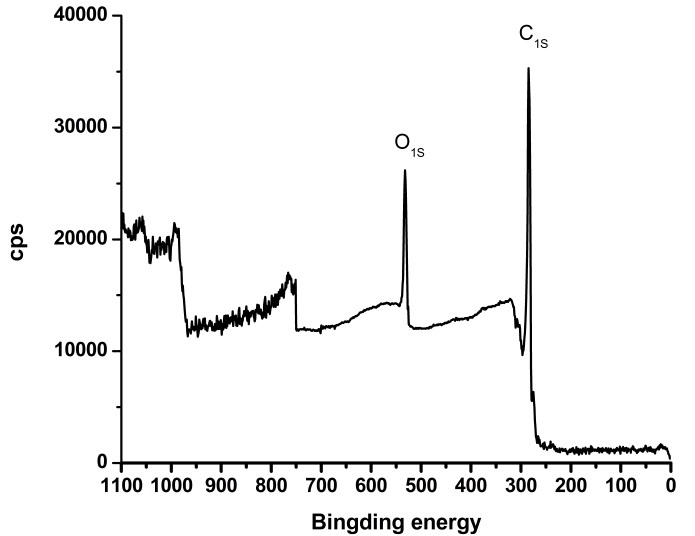
**

**Fig. S1.** The XPS analysis of oxidized carbon nanotubes

**Table S1** The elements content of carbon nanotules

| Peak | Type | Position BE (eV) | FWHM (eV) | Raw area cps (eV) | RSF | Atomic mass | Atomic cont % | Mass cont% |
| --- | --- | --- | --- | --- | --- | --- | --- | --- |
| Fe 2p | Reg | 716.950 | 0.070 | 27.6 | 2.947 | 55.846 | 0.01 | 0.05 |
| O 1S | Reg | 533.500 | 2.894 | 7741.5 | 0.736 | 15.999 | 14.85 | **18.85** |
| N 1S | Reg | 400.050 | 1.000 | 2.8 | 0.505 | 14.007 | 0.01 | 0.01 |
| C 1S | Reg | 284.950 | 1.159 | 19567.3 | 0.318 | 12.011 | 85.13 | 81.09 |

**Supplementary Fig. S2.**

**
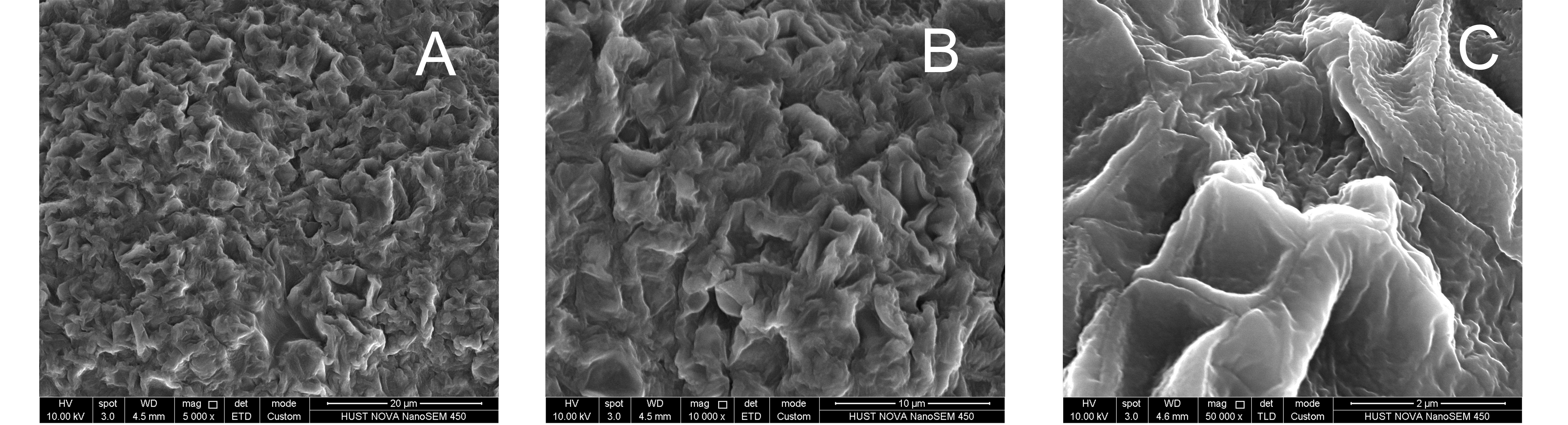
**

**Fig. S2.** **The SEM images of microcapsules surface. (a)** plotting scale: 20 μm; **(b)** plotting scale:10 μm; **(c)** plotting scale: 2 μm

**Supplementary Fig. S3.**

**
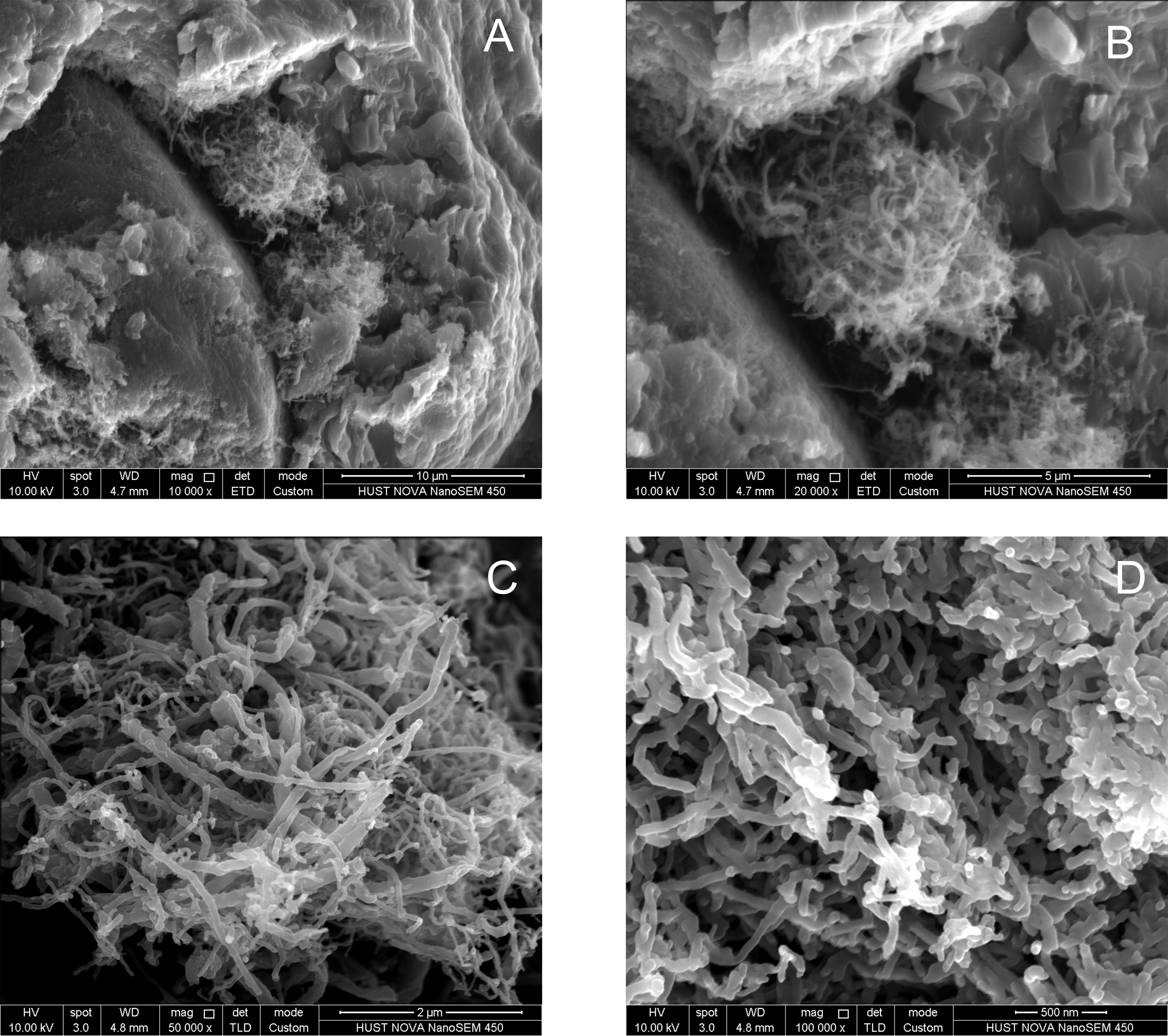
**

**Fig. S3.** **The SEM images of carbon nanotubes modified microcapsules. (a)** plotting scale:10 μm; **(b)** plotting scale:5 μm; **(c)** plotting scale:2 μm; **(d)** plotting scale: 500 nm.
